# Assessment of myocardial viability with [^15^O]water PET: A validation study in experimental myocardial infarction

**DOI:** 10.1007/s12350-019-01818-5

**Published:** 2019-07-17

**Authors:** Maria Grönman, Miikka Tarkia, Christoffer Stark, Tommi Vähäsilta, Tuomas Kiviniemi, Mark Lubberink, Paavo Halonen, Antti Kuivanen, Virva Saunavaara, Tuula Tolvanen, Jarmo Teuho, Mika Teräs, Timo Savunen, Mikko Pietilä, Seppo Ylä-Herttuala, Anne Roivainen, Juhani Knuuti, Antti Saraste

**Affiliations:** 1 Turku PET Centre, University of Turku, Turku, Finland; 2 Research Centre of Applied and Preventive Cardiovascular Medicine, University of Turku, Turku, Finland; 3 Heart Center, Turku University Hospital and University of Turku, Turku, Finland; 4 Department of Surgical Sciences, Uppsala University, Uppsala, Sweden; 5 Department of Medical Physics, Uppsala University Hospital, Uppsala, Sweden; 6 A.I. Virtanen Institute for Molecular Sciences, University of Eastern Finland, Kuopio, Finland; 7 Turku PET Centre, Turku University Hospital, Turku, Finland; 8 Department of Medical Physics, Turku University Hospital, Turku, Finland; 9 Heart Center, Kuopio University Hospital, Kuopio, Finland; 10 Turku Center for Disease Modeling, University of Turku, Turku, Finland; 11 Institute of Clinical Medicine, University of Turku, Turku, Finland

**Keywords:** Radiowater, PET, myocardial perfusion imaging, viability, myocardial infarction

## Abstract

**Background:**

Assessment of myocardial viability is often needed in patients with chest pain and reduced ejection fraction. We evaluated the performance of reduced resting MBF, perfusable tissue fraction (PTF), and perfusable tissue index (PTI) in the assessment of myocardial viability in a pig model of myocardial infarction (MI).

**Methods and results:**

Pigs underwent resting [^15^O]water PET perfusion study 12 weeks after surgical (n = 16) or 2 weeks after catheter-based (n = 4) occlusion of the proximal left anterior descending coronary artery. MBF, PTF, and PTI were compared with volume fraction of MI in matched segments as assessed by triphenyl tetrazolium chloride staining of LV slices. MBF and PTF were lower in infarcted than non-infarcted segments. Segmental analysis of MBF showed similar area under the curve (AUC) of 0.85, 0.86, and 0.90 with relative MBF, PTF, and PTI for the detection of viable myocardium defined as infarct volume fraction of < 75%. Cut-off values of relative MBF of ≥ 67% and PTF of ≥ 66% resulted in accuracies of 90% and 81%, respectively.

**Conclusions:**

Our results indicate that resting MBF, PTF, and PTI based on [^15^O]water PET perfusion imaging are useful for the assessment of myocardial viability.

**Electronic supplementary material:**

The online version of this article (10.1007/s12350-019-01818-5) contains supplementary material, which is available to authorized users.

## Introduction

Myocardium of patients with coronary artery disease (CAD) and left-ventricle (LV) dysfunction often contains a mixture of ischemic, but viable and irreversibly injured, non-viable tissue.^1^ Dysfunctional ischemic myocardium that is viable has the potential of regaining contractile function after revascularization.^2^,^3^ Thus, the assessment of myocardial viability is of importance in identifying patients with CAD and LV dysfunction that will most likely benefit from revascularization.^2^,^3^

[^15^O]water is a metabolically and chemically inert, freely diffusible tracer for positron emission tomography (PET) myocardial perfusion imaging that has been established for the quantification of myocardial blood flow (MBF) using one-tissue compartmental model.^4^ Current scanner and software technologies enable automated generation of myocardial perfusion images and reproducible quantification of MBF at segmental level to evaluate its regional distribution within coronary territories.^5^ Reduced MBF by [^15^O]water PET during vasodilator stress accurately detects regions of myocardial ischemia in patients with chest pain and suspected obstructive CAD.^6^-^9^

Differentiation between viable and non-viable myocardium by [^15^O]water PET is based on the concepts of reduced fraction of tissue that is capable of rapidly exchanging water^10^-^14^ as well as low and heterogeneous MBF^11^,^15^-^17^ in the injured myocardial regions. Thus, water-perfusable tissue fraction (PTF) that is defined as the fraction of tissue capable of rapidly exchanging [^15^O]water within a given volume of region of interest (ROI) as well as perfusable tissue index (PTI) that is the fraction of [^15^O]water-perfusable tissue within the total anatomical tissue fraction (ATF) within the ROI containing both perfusable and non-perfusable tissue components, become reduced in the absence of viable myocardium.^4^,^10^-^14^,^18^ Both PTF and PTI have been shown to predict the recovery of contractile function after revascularization in patients with acute^13^,^18^ or chronic myocardial infarction (MI).^10^,^12^,^14^,^17^ Resting MBF by [^15^O]water PET is reduced in areas of recent or chronic MI,^11^,^15^-^17^ but its value in the assessment of viability is not well defined. Thus, we evaluated resting MBF, PTF, and PTI assessed by [^15^O]water PET for the detection of infarcted and viable myocardium defined by 1% 2,3,5-triphenyltetrazolium chloride (TTC) staining in a pig model of MI.

## Methods

### Animals and Study Protocol

Three-month-old Finnish Landrace pigs were implanted a bottleneck stent in the proximal left anterior descending coronary artery (n = 4, weighing 28-39 kg) as described previously^19^ or underwent a 2-step occlusion of left anterior descending coronary artery with implantation of a proximal ameroid constrictor after distal ligation for the preconditioning of the heart (n = 16, weighing 88-130 kg).^20^,^21^ For this study, 20 pigs with a MI confirmed by TTC staining and PET myocardial perfusion imaging performed were retrospectively evaluated. Detailed protocol regarding anesthesia, stent implantation, and surgical operation is presented in the Supplemental methods. Myocardial perfusion was quantified using [^15^O]water PET as described below. Subsequently, the pigs were euthanized, and their hearts were excised for later analysis.

All animal experiments were approved by the national Animal Experiment Board in Finland and the Regional State Administrative Agency for Southern Finland and carried out in compliance with the EU legislation relating to the conduct of animal experimentation.

### PET Image Acquisition and Reconstruction

Pigs underwent the imaging 2 weeks after the stenting or 3 months after the surgical operation. All the animals underwent a myocardial perfusion PET study with [^15^O]water at rest as previously described^21^ with a Discovery 690 hybrid PET/CT scanner (GE Medical Systems, Milwaukee, WI, USA). [^15^O]water (680 ± 160 MBq) was injected as an intravenous (i.v.) bolus over 15 seconds at an infusion rate of 10 ml/min via the ear vein. A dynamic scanning of 4 minutes 40 seconds (time frames 14 × 5 seconds, 3 × 10 seconds, 3 × 20 seconds and 4 × 30 seconds) was performed. The acquired [^15^O]water PET data were corrected for attenuation, scatter, random counts, and dead time, and reconstructed with an iterative VUE Point algorithm using two iterations and 24 subsets. The whole transaxial field of view (70 cm) was reconstructed in 128 × 128 matrix yielding pixel size of 5.47 mm × 5.47 mm. The device produces 47 axial planes with a slice thickness of 3.27 mm.

### TTC Staining

Immediately after the PET scanning, the animals were sacrificed by an i.v. injection of potassium chloride (B. Braun Medical Oy, Helsinki, Finland). The heart was excised, the LV was cut into four short axis slices from base to apex that were incubated for 15 minutes in 1% TTC (Sigma-Aldrich, Saint Louis, MO, USA), diluted in phosphate-buffered saline (pH 7.4) at 37 °C, and photographed from both sides. In two pigs, only the mid-cavity part of the TTC-stained LV was available.

The infarcted tissue was defined in the TTC photographs visually. The heart was divided into segments using a standard 17 segments division. The volume fraction of the infarct in each segment was categorized as follows: 0: no infarct; 1: infarct size < 25%; 2: 25%-49%; 3: 50%-74%, and 4: ≥ 75% of the size of the segment. Non-viable myocardium was defined as infarct fraction ≥ 75% or ≥ 50%. The apical segment 17 was excluded from the analyses.

### PET Image Analyses

PET images were analyzed with Carimas v2.9 software (Turku PET Centre, Turku, Finland, http://turkupetcentre.fi/carimas/) using Heart tool as described earlier.^21^ The long axis of LV was defined manually, myocardial contours were first delineated semi-automatically and, if necessary, modified manually, and finally volume of interest (VOI) covering the whole LV myocardium was applied.

The PTF and absolute segmental LV MBF was quantified from [^15^O]water images as ml/min/g using a single-compartment model described earlier.^22^,^23^ The PET measurements were displayed as polar maps. To obtain the relative MBF values, the polar maps of [^15^O]water at rest were normalized to mean value of four posterolateral segments (segments 4, 5, 10, and 11). The infarcted area in every pig was in the anterior region subtended by left anterior coronary artery thus leaving the posterolateral region outside the ischemic area. If one of these segments was infarcted according to TTC, it was excluded from the mean value. In order to assess reproducibility of measurements, the same observer repeated analyses twice, and within-subject coefficients of variations were calculated by using root-mean-square approach. Coefficients of variations for repeated measurements of absolute MBF, relative MBF, and PTF measurements were 6% in the remote non-infarcted segments. In segments with infarct volume fraction ≥ 75%, coefficients of variations were 19%, 18%, and 10%, respectively.

Since the thickness of the LV wall may be reduced in the infarcted myocardium, effect of ROI thickness on absolute MBF, relative MBF, and PTF was evaluated by manually increasing or reducing ROI thickness from default. In further analyses, ROI thickness was manually reduced to completely fit inside the myocardium in the CT images in order to account for possible reduced wall thickness in the infarcted myocardium.

In order to compare MBF and PTF in viable and non-viable myocardium, their circumferential profiles were analyzed in the midventricular level in 48 sectors in pigs that had transmural infarction. Values were compared in the transmurally infarcted area (average of three adjacent sectors) and remote areas (average of six sectors).

In order to obtain PTI measurements, pigs were further analyzed with aQuant software (MedTrace Pharma A/S, Denmark) as described earlier.^24^ For technical reasons, only 11 pigs were included in PTI measurements.

### Statistical Analyses

All data are expressed as mean ± SD. SPSS Statistics software v. 25 (IBM, NY, USA) was used for statistical analyses. Receiver operating characteristic (ROC) analyses were performed, Youden index was used to obtain optimal cut-off values for [^15^O]water PET in comparison to TTC staining and the method of DeLong^25^ was used to compare area under the curve (AUC) values. Ability of the PET parameters to distinguish the infarcted areas of different severity and viable areas from the non-infarcted areas was tested.

## Results

### Myocardial Infarction and Hemodynamics

**Figure 1:**
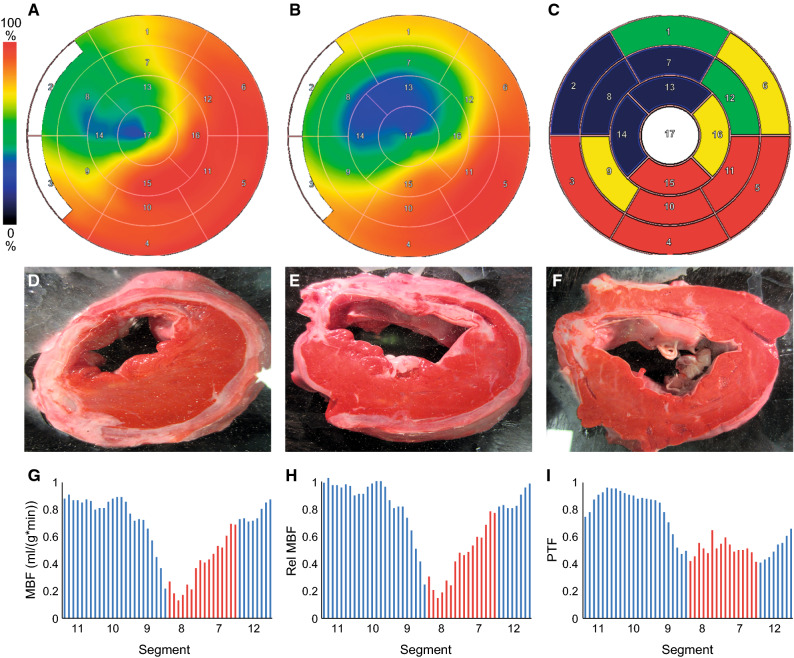
Representative polar maps of resting myocardial blood flow (MBF) by [^15^O]water PET (**A**) and perfusable tissue fraction (PTF) by [^15^O]water PET (**B**). Polar maps were normalized to their own maximum. (**C**) shows grading of segmental volume fraction of myocardial infarction (red = 0%, yellow = 1%-24%, green = 25%-49%, and blue = > 75%) based on TTC staining of myocardial slices at the apical (**D**), papillary muscle (**E**), and basal (**F**) levels. Viable myocardium is stained red and non-viable infarcted white by TTC. MBF and PTF are reduced in segments with myocardial infarction. (**G**), (**H**), and (**I**) show examples of circumferential profiles of MBF, relative MBF, and PTF, respectively. Sectors with transmural infarction are marked with red color

Twenty pigs had MI defined by TTC staining and were included in the study. An example of TTC staining of MI in myocardial tissue slices representing the apical, papillary muscle, and basal levels is shown in Figure 1.

Transmural infarct scar was detected in 14 pigs. Based on TTC staining, number of segments without MI was 152, whereas 54 segments showed MI volume fraction of < 25%, 37 25 - < 50%, 26 50 - < 75%, and 41 ≥ 75%. Thus, 41 segments with ≥ 75% of infarction were defined as non-viable and 269 segments with <75% of infarction were defined as viable.

The average heart rate of pigs at rest was 91 ± 18 bpm, systolic blood pressure 135 ± 24 mmHg, and rate pressure product 12 000 ± 3 300 mmHg bpm.

### MBF and PTF in Myocardial Infarction

Figure 1 shows examples of polar maps of regional MBF and PTF measured by [^15^O]water together with TTC-stained slices of the LV, and examples of circumferential profiles of MBF and PTF (Figure 1). Compared with the remote myocardium, transmural infarction showed lower absolute MBF (0.45 ± 0.34 vs. 1.23 ± 0.47, *P* < 0.001), relative MBF (0.37 ± 0.23 vs. 0.99 ± 0.014, *P* < 0.001), and PTF (0.55 ± 0.12 vs. 84 ± 0.11, *P* < 0.001).

Segmental absolute MBF, relative MBF, and PTF by [^15^O]water PET in relation to segmental volume fraction of infarction are presented in Table 1. Overall, segmental absolute and relative MBF as well as segmental PTF by [^15^O]water PET gradually lowered in the presence of increasing infarction volume fraction in the segment. Compared with non-infarcted segments, absolute and relative MBF were lower in segments with any infarction, whereas PTF was lower only when infarct volume fraction was ≥ 50%. In segments defined as non-viable based on infarct volume fraction ≥ 75%, absolute and relative MBF as well as PTF were lower than in any segments with infarct fraction < 75%.

**Table 1:** Segmental resting absolute myocardial blood flow (MBF), relative MBF, and perfusable tissue fraction (PTF) by [^15^O]water PET in relation to segmental infarct volume fraction

Infarct volume fraction (%)	Number of segments	MBF [ml/(g·min)]	Relative MBF	PTF
0	152	1.11 (± 0.38)	0.99 (± 0.19)	0.81 (± 0.13)
1–24	54	0.95 (± 0.33)*	0.87 (± 0.21)*	0.76 (± 0.17)
25–49	37	0.88 (± 0.28)*	0.83 (± 0.20)*	0.77 (± 0.16)
50–74	26	0.79 (± 0.23)*	0.83 (± 0.22)*	0.68 (± 0.23)*
≥ 75	41	0.55 (± 0.27)*,**	0.56 (± 0.31)*,**	0.51 (± 0.19)*,**

*P < 0.05 vs. no infarction, **P < 0.05 vs. infarct volume 0%, 1–24%, 25–49% or 50–74%

**Figure 2:**
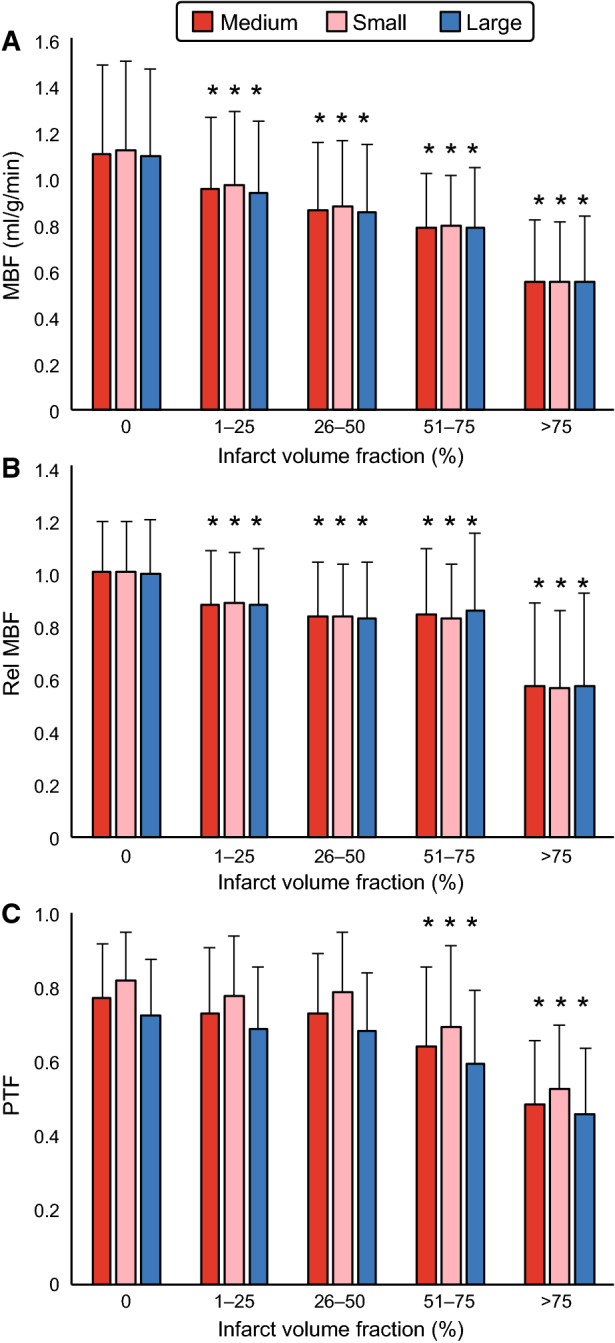
Segmental values of absolute myocardial blood flow (MBF, **A**), relative MBF (**B**), and perfusable tissue fraction (PTF, **C**) by [^15^O]water PET measured using three different ROI thickness: medium = ROI width defined automatically by the software program, small = ROI width manually reduced to completely fit inside the myocardium, large = ROI width manually increased from the medium ROI. *P < 0.05 vs. segments with infarct volume fraction 0

The effect of ROI thickness on segmental absolute MBF, relative MBF, and PTF by [^15^O]water PET is presented in Figure 2. ROI thickness had no effect on absolute or relative MBF. An increase in ROI thickness, however, resulted in a decrease in PTF values. There was an average difference of 12% ± 6% (*P* < 0.001) between small and large ROI thickness in segments with infarct fraction of < 75% and 13% ± 10% (*P* = 0.29) in segments with infarct fraction of ≥ 75%.

### Performance of Segmental MBF and PTF in the Detection of Infarction

**Figure 3:**
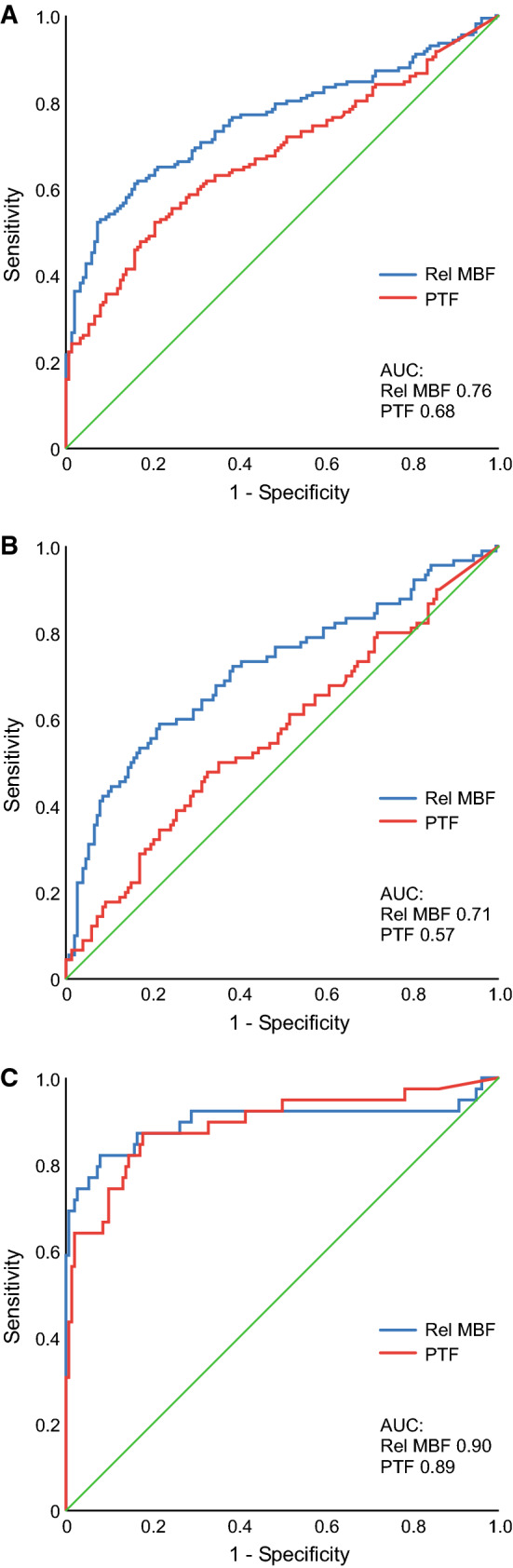
Receiver operating characteristics (ROC) curve analysis of segmental relative myocardial blood flow (Rel MBF) and perfusable tissue fraction (PTF) by [^15^O]water PET in the detection of any myocardial infarction in the corresponding segment (**A**), subendocardial infarction (infarct volume fraction 1%-49%, **B**), or non-viable tissue (infarct volume fraction ≥ 75%, **C**)

Figure 3 shows the results of ROC analysis of segmental relative MBF and PTF by [^15^O]water PET in the detection of MI in the corresponding segment.

We first evaluated the performance of segmental relative MBF and PTF in the detection of any MI (segments with any infarction vs. no infarction). Relative MBF of ≤ 85% and PTF of ≤ 70% were the optimal cut-off values for the detection of any MI demonstrating modest sensitivity, but high specificity and positive predictive value. Relative MBF was more accurate than PTF in detecting any MI (AUC 0.76 vs. 0.68, *P* = 0.04). We further evaluated the performance of segmental relative MBF and PTF in the detection of either subendocardial infarction (segments with infarct volume fraction 1%-49% vs. no infarction) or non-viable tissue (infarct volume fraction ≥ 75% vs. no infarction). Based on the ROC analysis, relative MBF performed better in the detection of subendocardial infarction than PTF (AUC 0.71 vs. 0.57, *P* = 0.004). There was no difference, however, between relative MBF and PTF in the detection of non-viable tissue defined as ≥ 75% infarct volume fraction (AUC 0.90 vs. 0.89, *P* = 0.89).

Using the cut-off values derived from the ROC curve analysis, average size of the transmural MI of the LV was 29% ± 15% of the LV as measured by [^15^O]water PET with relative MBF and 29% ± 22% as measured by [^15^O]water PET with PTF (*P* = 0.99).

### Performance of Segmental MBF and PTF in the Assessment of Myocardial Viability

**Figure 4:**
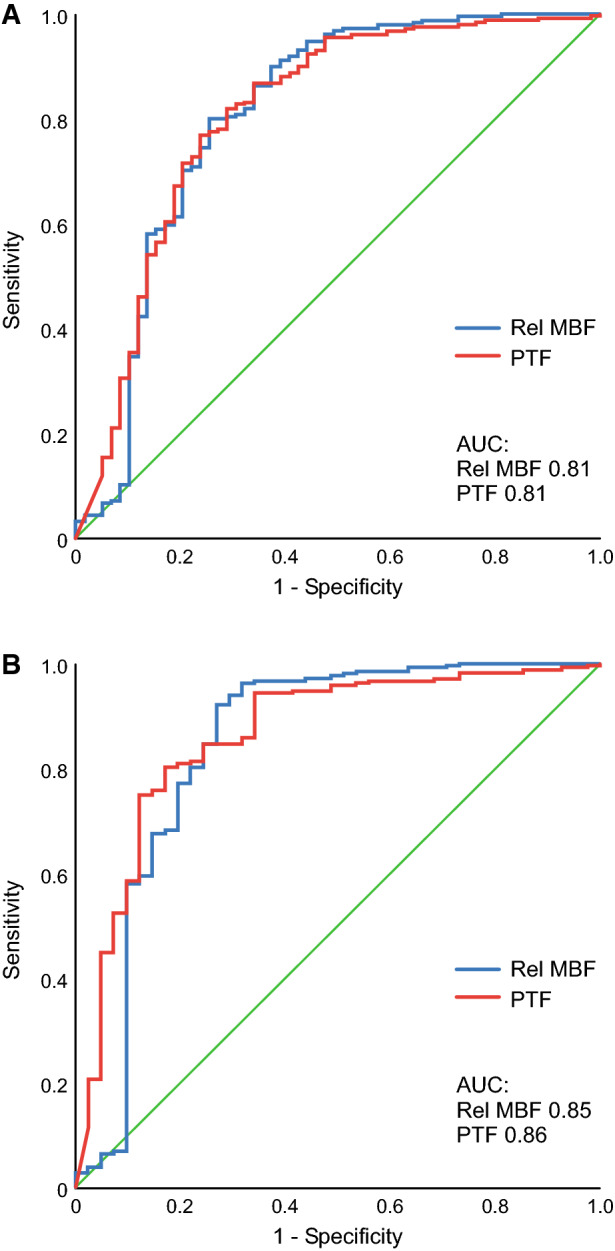
Receiver operating characteristics (ROC) curve analysis of relative myocardial blood flow (Rel MBF) and perfusable tissue fraction (PTF) by [^15^O]water in the detection of myocardial viability with infarct volume fraction of < 50% (**A**) and with infarct volume fraction of < 75% (**B**)

For the assessment of viability, the segments with infarct volume fraction of < 50% were defined as viable and segments with infarct volume fraction of ≥ 50% as non-viable. ROC analysis showed AUC of 0.81 with relative MBF and 0.81 with PTF for the detection of viability (*P* = 0.97) (Figure 4). Relative MBF ≥ 79% and PTF ≥ 66% were the optimal cut-off values for the detection of viable myocardium showing similar sensitivities, specificities, and diagnostic accuracies (Table 2). Previously, PTF ≥ 70% has been shown as the optimal cut-off value for the detection of viability.^26^ Although close to the cut-off value determined in this study, it resulted in lower diagnostic accuracy in our material (Table 2).

**Table 2:** Performance of segmental relative myocardial blood flow (Rel MBF) and perfusable tissue fraction (PTF) by [^15^O]water in the assessment of myocardial infarction and viability

	Sensitivity	Specificity	Positive predictive value	Negative predictive value	Accuracy
No infarction vs. any infarction					
Rel MBF (cut-off ≤ 85%)	61%	84%	80%	67%	72%
PTF (cut-off ≤ 70%)	54%	79%	76%	61%	66%
Viable tissue (infarct volume fraction < 50%)					
Rel MBF (cut-off ≥ 79%)	81%	69%	92%	46%	79%
PTF (cut-off ≥ 66%)	82%	71%	92%	48%	80%
PTF (cut-off ≥ 70%)	74%	76%	93%	41%	74%
Viable tissue (infarct volume fraction < 75%)					
Rel MBF (cut-off ≥ 67%)	92%	73%	96%	59%	90%
PTF (cut-off ≥ 66%)	80%	83%	97%	39%	81%
PTF (cut-off ≥ 70%)	72%	88%	97%	32%	74%

Because the recovery of function in the presence of infarct volume fraction of 50%-75% may be obscure, we also evaluated the performance of relative MBF and PTF in the detection of viability defining segments with infarct volume fraction of ≥ 75% as non-viable. The analysis showed AUC of 0.85 with relative MBF and 0.86 with PTF (*P* = 0.77) (Figure 4). Relative MBF ≥ 67% and PTF ≥ 66% were the optimal cut-off values for the detection viable myocardium resulting in accuracy of 90% and 81%, respectively (Table 2).

### Comparison of Segmental MBF, PTF, and PTI in the Assessment of Myocardial Viability

**Figure 5:**
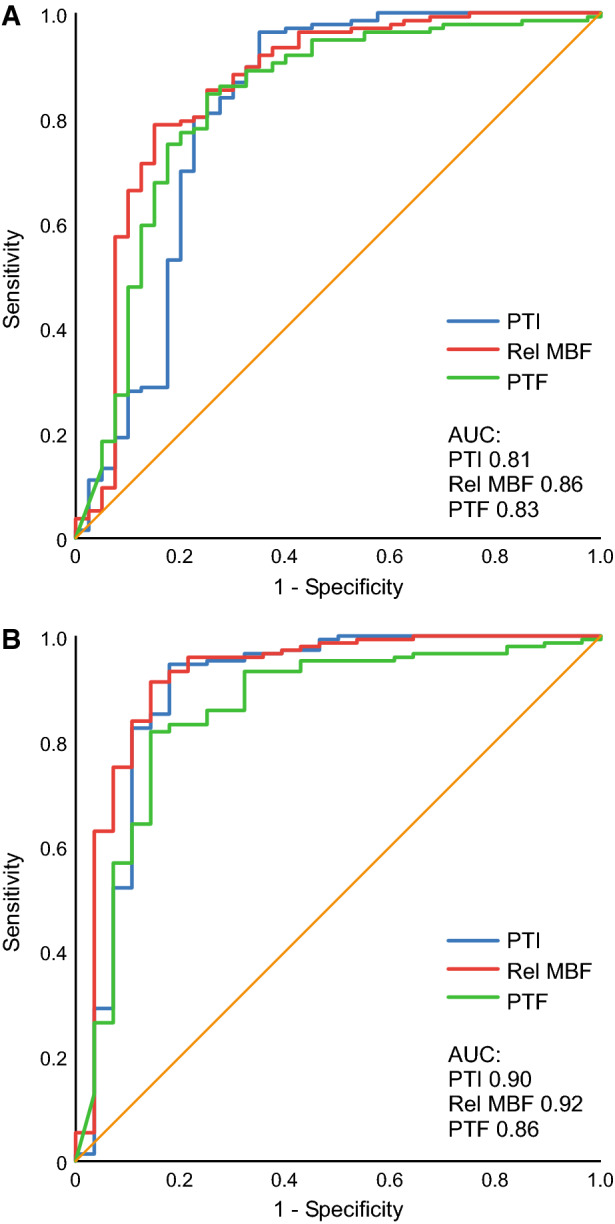
Receiver operating characteristics (ROC) curve analysis of relative myocardial blood flow (Rel MBF), perfusable tissue fraction (PTF), and perfusable tissue index (PTI) by [^15^O]water in the detection of myocardial viability with infarct volume fraction of < 50% (**A**) and with infarct volume fraction of < 75% (**B**)

Segmental MBF and PTF were compared with PTI for the detection of viability in 11 pigs. The ROC analysis showed AUC of 0.81 with PTI for the detection of viability defined as segments with infarct volume fraction of < 50% (Figure 5). There was no difference between PTI and relative MBF (*P* = 0.41) or PTF (*P* = 0.79). When viability was defined as segments with infarct volume fraction of < 75%, ROC analysis showed AUC of 0.90 with PTI. There was no difference between PTI and relative MBF (*P* = 0.67) or PTF (*P* = 0.50). The optimal cut-off value of PTI was ≥ 82% for the detection of viability, which yielded slightly better sensitivity, specificity, and diagnostic accuracy than those of relative MBF or PTF (Supplemental Table).

## Discussion

We evaluated the performance of resting MBF, PTF, and PTI by [^15^O]water PET in the detection of MI and viability in comparison with histology in a pig model. The main findings of the present study are that both MBF and PTF are reduced in myocardial regions with chronic infarction, and relative MBF, PTF, and PTI all perform well in the detection of non-viable myocardium. In comparison to PTF, MBF appeared non-dependent on ROI thickness and it is reduced already in the presence of small infarct volume fraction. Our results in this model indicate that resting MBF as well as PTF and PTI by [^15^O]water PET are both useful indicators of impaired myocardial viability. Their optimal cut-off values, accuracy, and performance in predicting functional outcomes after revascularization, however, need to be tested in clinical studies.

Both PTF and PTI have been shown to predict recovery of contractile function after revascularization in patients with MI with threshold values of at least 0.7 of the remote region.^10^,^12^-^14^,^17^,^18^ Our results are comparable to previous studies indicating that segmental PTF < 0.66 and PTI < 0.82 accurately detected non-viable myocardial segments defined as infarct volume fraction of ≥ 75% in the corresponding segment. The original model measures MBF in water-exchanging myocardium and is independent of ROI thickness.^4^,^22^,^23^ Our study showed similar results on MBF. As expected, an increase in ROI thickness, however, reduced PTF and therefore, careful standardization of ROI definition is required. Alternatively, PTI may be used that is calculated as the ratio of PTF and ATF which contains both perfusable and non-perfusable tissue components.^4^,^18^ Determining ATF, however, requires blood-pool imaging with [^15^O]CO or a software-based calculation of parametric PTI images from single [^15^O]water scan.^24^,^26^

Resting MBF by [^15^O]water PET is reduced in the presence of an MI scar both in experimental models^15^,^16^,^27^ and in patients.^10^,^17^,^28^ In experimental models, relatively good agreement between [^15^O]water PET and microspheres has been found at the center of infarct scar area.^15^,^16^ In our study, MBF in the center of transmural infarction was 0.45 ± 0.34 ml/g/min that is in agreement with previous studies reporting MBF of 0.35 ± 0.34 ml/g/min,^16^ 0.38 ± 0.17 ml/g/min,^15^ or 0.45 ± 0.11 ml/g/min.^17^ In order to account for variability in global resting MBF in pigs with MI scar, we also determined relative MBF normalized for flow in the remote myocardium in detection of non-viable segments. Overestimation of MBF by [^15^O]water PET has been found in low-flow regions when larger ROI size including a mixture of infarcted and viable areas is used.^16^,^28^ This may be explained by measuring perfusion in the PTF that is inversely related to the extent of infarcted tissue in the ROI.^11^ We found that MBF values were not influenced by thickness of the ROI, but we found higher segmental MBF values (on average 0.55 ml/g/min) than values at the center of infarction even in those segments that were defined as non-viable (i.e., infarct fraction ≥ 75%).

For practical reasons, we focused on the value of MBF, PTF, and PTI in predicting MI and viability on segmental level. Sensitivities of relative MBF, PTF, and PTI were relatively low in detecting any MI. We found, however, that relative MBF below threshold of 67%, PTF below 66%, and PTI below 82% were associated with the absence of viability defined as infarct fraction of ≥ 75% in the corresponding segment. MBF, PTF, and PTI showed comparable accuracies in detecting viability based on the ROC analysis (AUC of 0.85, 0.86, and 0.90, respectively). MBF had particularly high sensitivity (90%) for detecting viable segments, with somewhat lower specificity probably reflecting areas of normal MBF in the presence of subendocardial infarction. Comparison with other imaging modalities indicates higher or similar accuracy with relative MBF (79%-90%) in our study than with [^18^F]FDG PET (79%), dobutamine echocardiography (79%), [^99m^Tc]sestamibi SPECT (75%), [^201^Th] SPECT (71%), and magnetic resonance imaging (MRI) techniques (61%-78%) with the endpoint of improvement of regional function.^3^ MBF, PTF, and PTI, however, are readily available in resting [^15^O]water PET perfusion studies and thus, may have the potential to serve as initial markers of viability guiding further assessment with other modalities.

## Study Limitations

There are some limitations that should be acknowledged. In clinical practice, assessment of both myocardial ischemia and viability is of importance and is performed in combination. However, in the setting of MI scar and well-developed collaterals^19^ in the pig model, we performed [^15^O]water PET only at rest. Reproducibility of MBF and PTF measurements was lower in the infarcted than non-infarcted segments that may be related to small absolute values in the infarcted segments. Although the average resting flow values in pigs were comparable to those measured in man, they are dependent on hemodynamic conditions that varied in anesthetized animals and therefore, the cut-off values as well as respective accuracies need to be determined in patients with MI. Due to concerns related to species differences and effects of anesthesia, we evaluated cut-off values for relative MBF rather than absolute MBF for the detection of infarction and viability. The clinical endpoint in viability studies is recovery of contractile function after revascularization and this needs to be assessed in future clinical studies. However, our experimental study provides evidence that evaluation of viability by [^15^O]water PET is feasible.

## New Knowledge Gained

Our results indicate that both resting MBF and PTF based on [^15^O]water PET imaging detect segments with MI scar and can serve as markers of myocardial viability that are readily available.

## Conclusions

Our results in this experimental model of MI indicate that resting MBF, PTF, and PTI based on [^15^O]water PET perfusion imaging are useful for the assessment of myocardial viability.

## Electronic supplementary material

Below is the link to the electronic supplementary material.

Supplementary material 1 (PPTX 670 kb)

Supplementary material 2 (DOCX 25 kb)

## Abbreviations

AUC: Area under the curve
CAD: Coronary artery disease
LV: Left ventricle
MBF: Myocardial blood flow
MI: Myocardial infarction
PET: Positron emission tomography
PTF: Perfusable tissue fraction
PTI: Perfusable tissue index
ROI: Region of interest
TTC: Triphenyl tetrazolium chloride
