# Supplementary material for: Assessment of myocardial viability with [15O]water PET: A validation study in experimental myocardial infarction
Source: J Nucl Cardiol. 2019 Jul 17;28(4):1271–80. doi: 10.1007/s12350-019-01818-5 (PMC8421281; doi:10.1007/s12350-019-01818-5)
Supplement: Supplementary file 1 — Supplementary material 1 (PPTX 670 kb) [file 12350_2019_1818_MOESM1_ESM.pptx]

## Slide 1
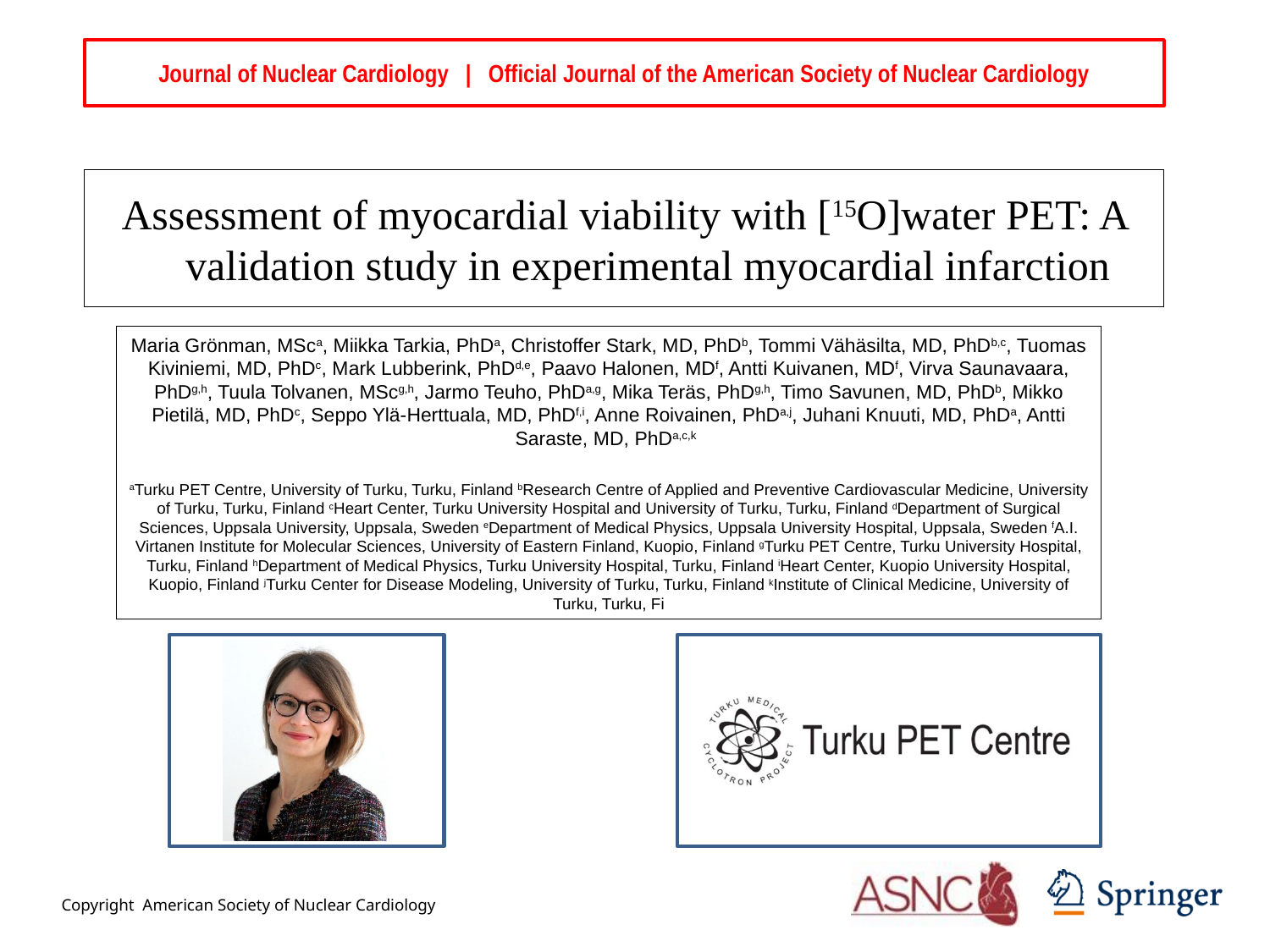

Journal of Nuclear Cardiology | Official Journal of the American Society of Nuclear Cardiology
# Assessment of myocardial viability with [15O]water PET: A validation study in experimental myocardial infarction
Maria Grönman, MSca, Miikka Tarkia, PhDa, Christoffer Stark, MD, PhDb, Tommi Vähäsilta, MD, PhDb,c, Tuomas Kiviniemi, MD, PhDc, Mark Lubberink, PhDd,e, Paavo Halonen, MDf, Antti Kuivanen, MDf, Virva Saunavaara, PhDg,h, Tuula Tolvanen, MScg,h, Jarmo Teuho, PhDa,g, Mika Teräs, PhDg,h, Timo Savunen, MD, PhDb, Mikko Pietilä, MD, PhDc, Seppo Ylä-Herttuala, MD, PhDf,i, Anne Roivainen, PhDa,j, Juhani Knuuti, MD, PhDa, Antti Saraste, MD, PhDa,c,k
aTurku PET Centre, University of Turku, Turku, Finland bResearch Centre of Applied and Preventive Cardiovascular Medicine, University of Turku, Turku, Finland cHeart Center, Turku University Hospital and University of Turku, Turku, Finland dDepartment of Surgical Sciences, Uppsala University, Uppsala, Sweden eDepartment of Medical Physics, Uppsala University Hospital, Uppsala, Sweden fA.I. Virtanen Institute for Molecular Sciences, University of Eastern Finland, Kuopio, Finland gTurku PET Centre, Turku University Hospital, Turku, Finland hDepartment of Medical Physics, Turku University Hospital, Turku, Finland iHeart Center, Kuopio University Hospital, Kuopio, Finland jTurku Center for Disease Modeling, University of Turku, Turku, Finland kInstitute of Clinical Medicine, University of Turku, Turku, Fi
Copyright American Society of Nuclear Cardiology

## Slide 2
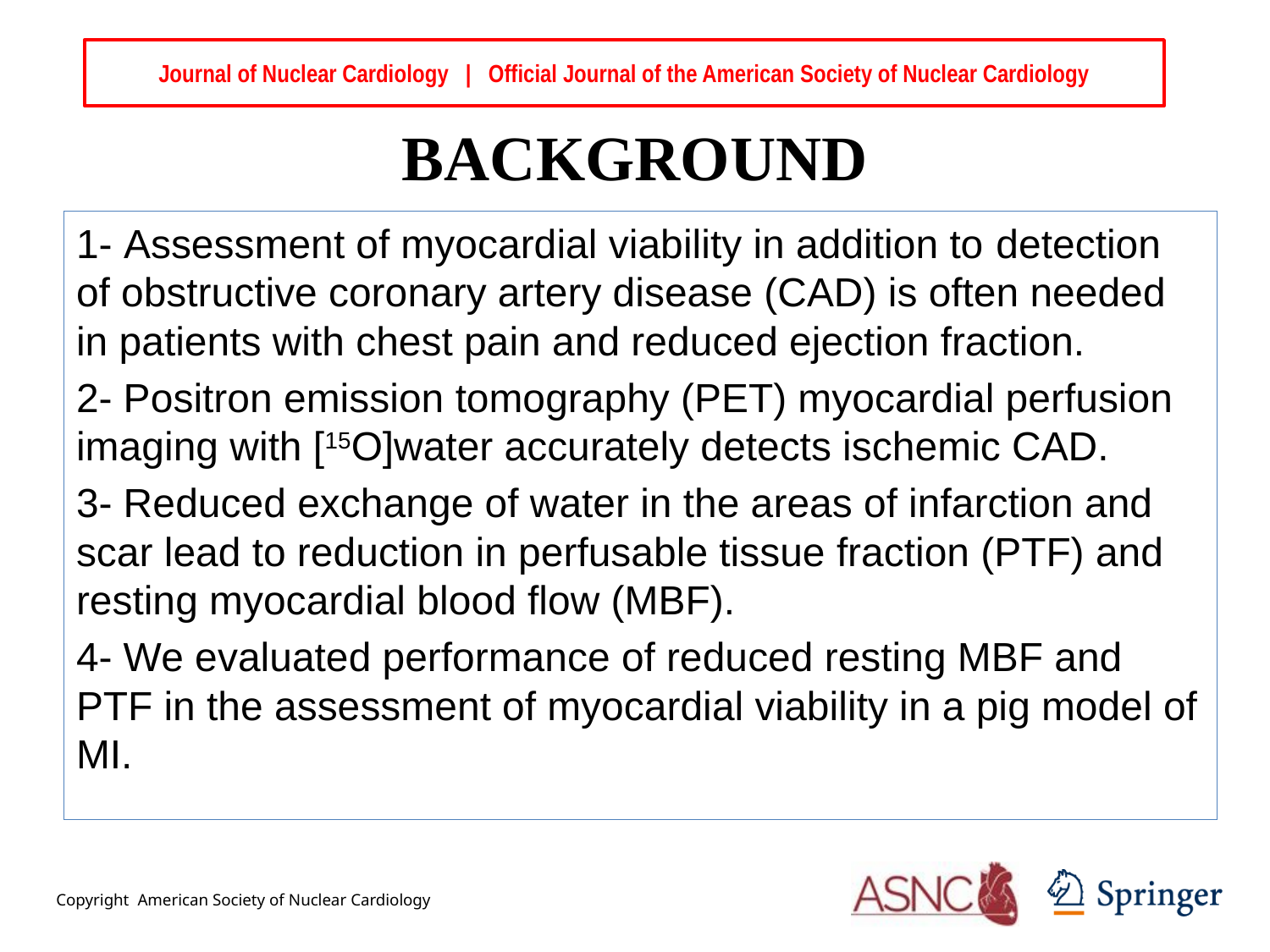

Journal of Nuclear Cardiology | Official Journal of the American Society of Nuclear Cardiology
# BACKGROUND
1- Assessment of myocardial viability in addition to detection of obstructive coronary artery disease (CAD) is often needed in patients with chest pain and reduced ejection fraction.
2- Positron emission tomography (PET) myocardial perfusion imaging with [15O]water accurately detects ischemic CAD.
3- Reduced exchange of water in the areas of infarction and scar lead to reduction in perfusable tissue fraction (PTF) and resting myocardial blood flow (MBF).
4- We evaluated performance of reduced resting MBF and PTF in the assessment of myocardial viability in a pig model of MI.
Copyright American Society of Nuclear Cardiology

## Slide 3
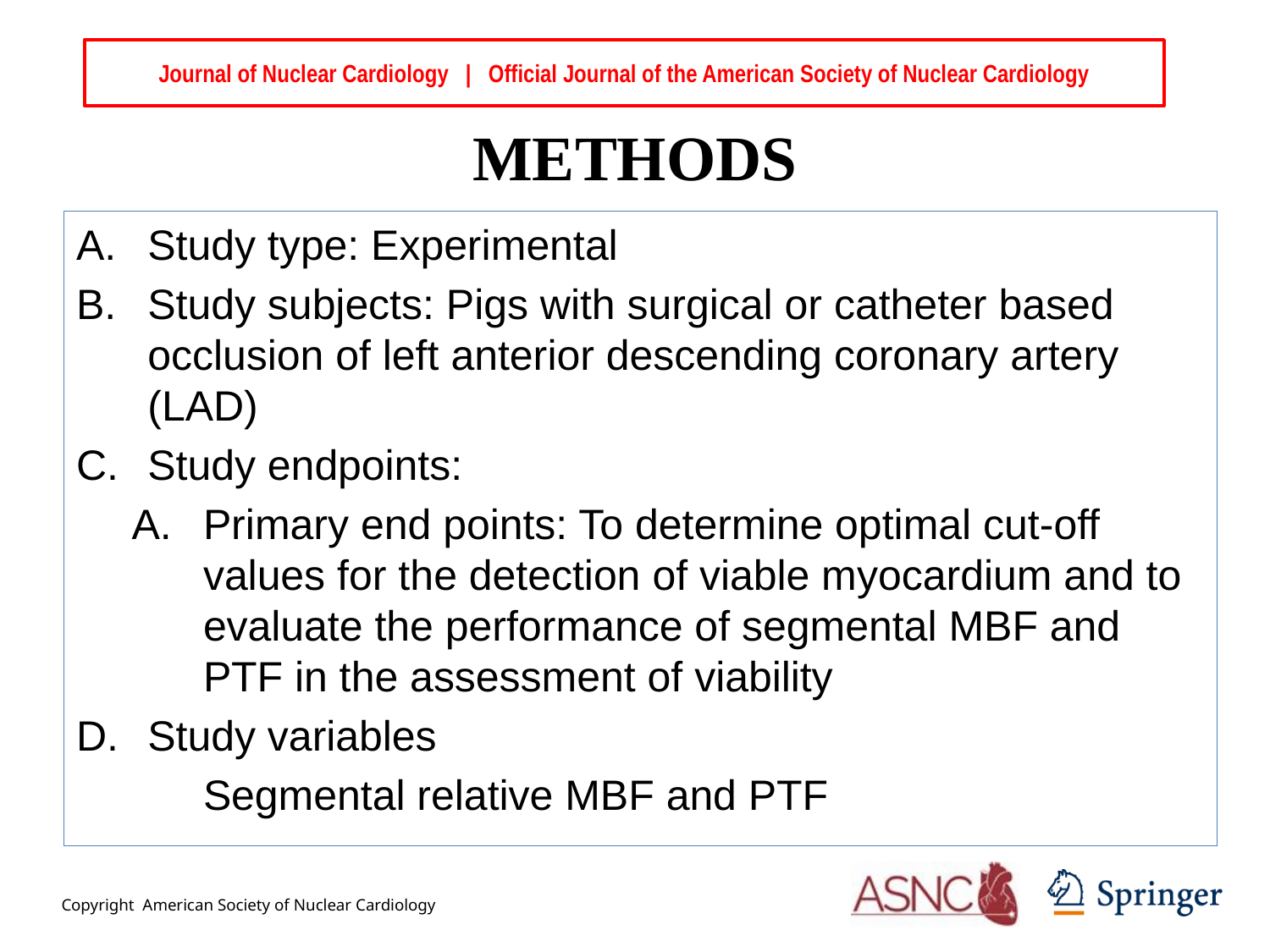

Journal of Nuclear Cardiology | Official Journal of the American Society of Nuclear Cardiology
# METHODS
Study type: Experimental
Study subjects: Pigs with surgical or catheter based occlusion of left anterior descending coronary artery (LAD)
Study endpoints:
Primary end points: To determine optimal cut-off values for the detection of viable myocardium and to evaluate the performance of segmental MBF and PTF in the assessment of viability
Study variables
	Segmental relative MBF and PTF
Copyright American Society of Nuclear Cardiology

## Slide 4
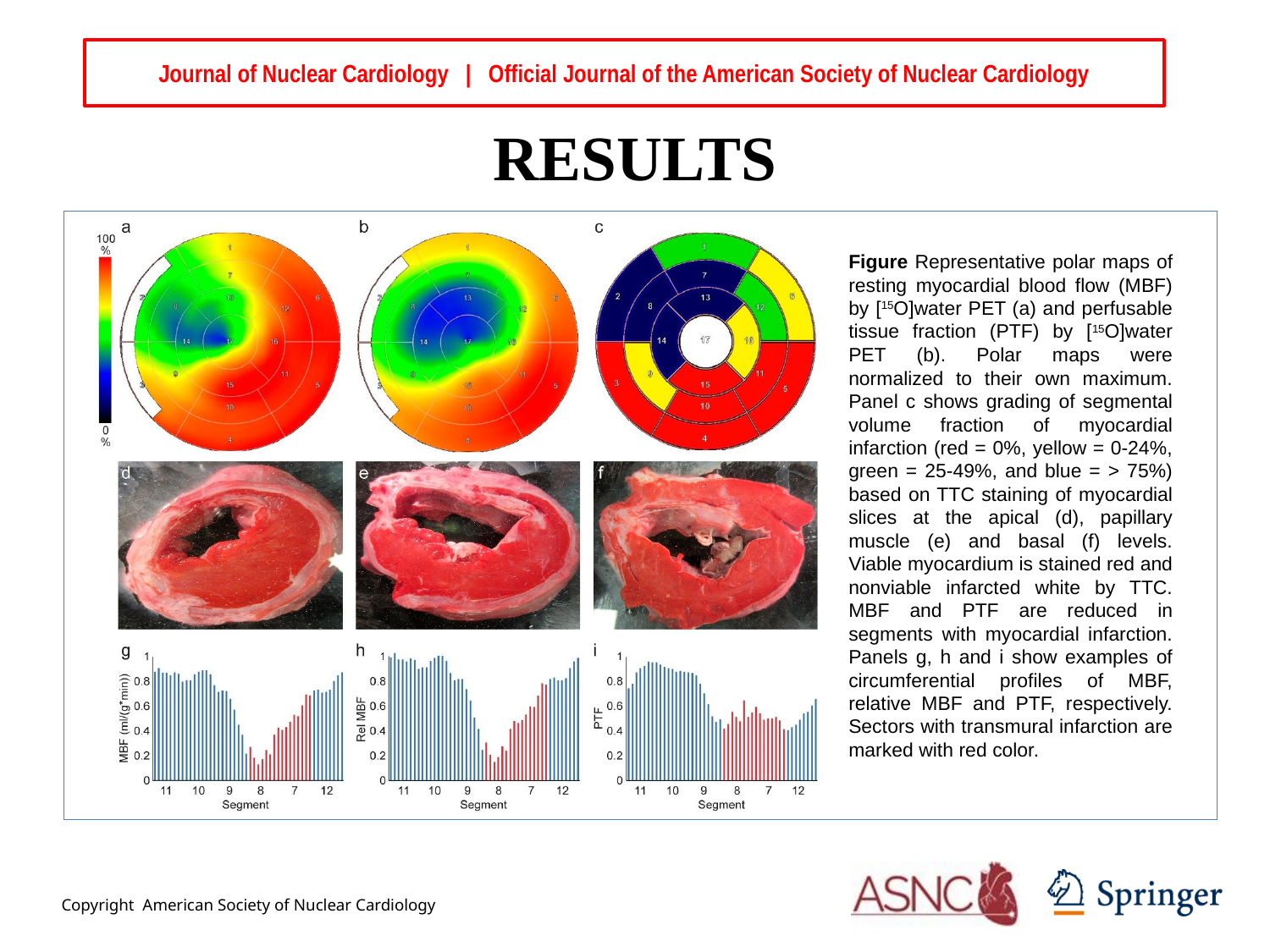

Journal of Nuclear Cardiology | Official Journal of the American Society of Nuclear Cardiology
# RESULTS
Figure Representative polar maps of resting myocardial blood flow (MBF) by [15O]water PET (a) and perfusable tissue fraction (PTF) by [15O]water PET (b). Polar maps were normalized to their own maximum. Panel c shows grading of segmental volume fraction of myocardial infarction (red = 0%, yellow = 0-24%, green = 25-49%, and blue = > 75%) based on TTC staining of myocardial slices at the apical (d), papillary muscle (e) and basal (f) levels. Viable myocardium is stained red and nonviable infarcted white by TTC. MBF and PTF are reduced in segments with myocardial infarction. Panels g, h and i show examples of circumferential profiles of MBF, relative MBF and PTF, respectively. Sectors with transmural infarction are marked with red color.
Copyright American Society of Nuclear Cardiology

## Slide 5
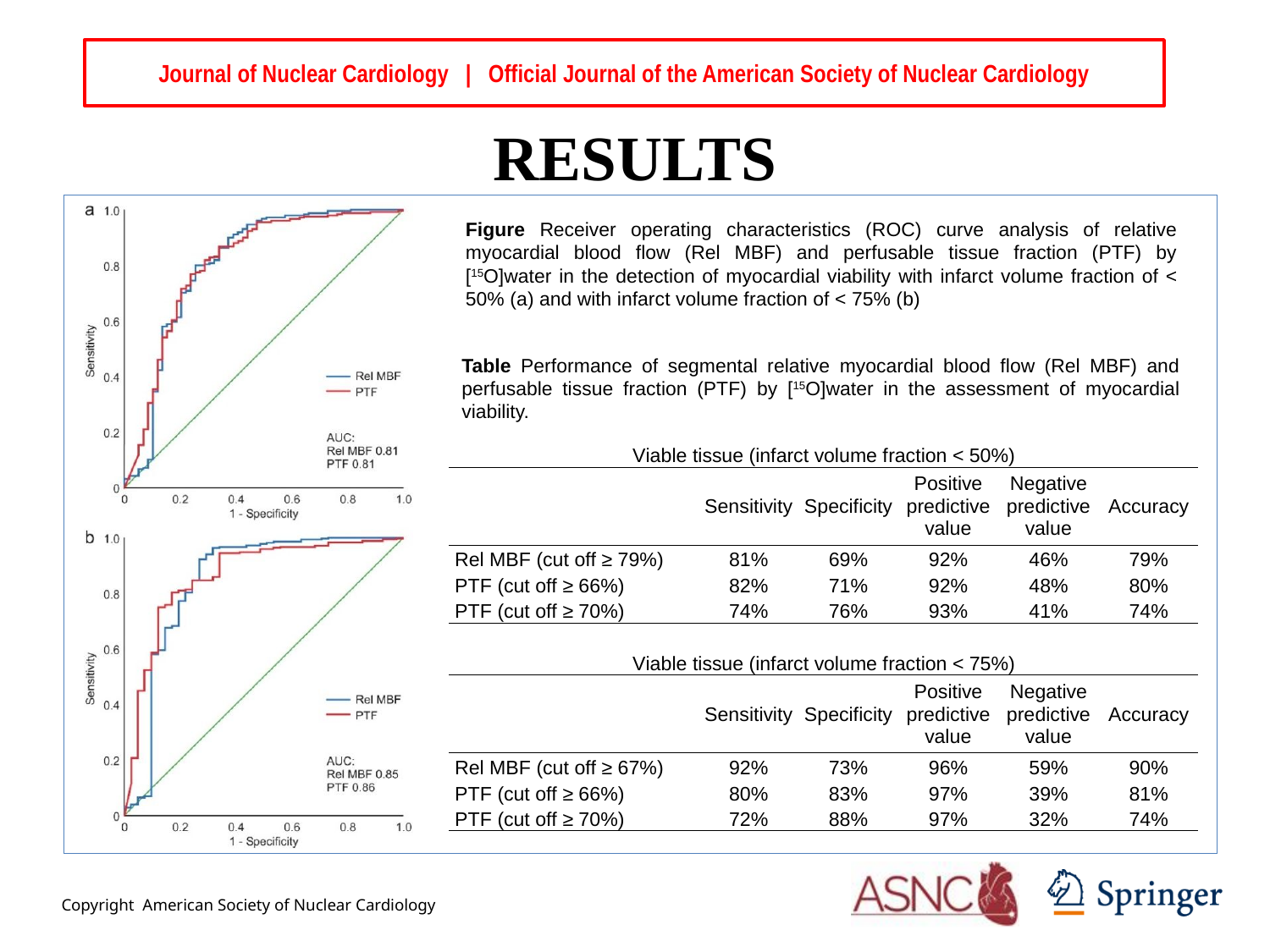

Journal of Nuclear Cardiology | Official Journal of the American Society of Nuclear Cardiology
# RESULTS
Figure Receiver operating characteristics (ROC) curve analysis of relative myocardial blood flow (Rel MBF) and perfusable tissue fraction (PTF) by [15O]water in the detection of myocardial viability with infarct volume fraction of < 50% (a) and with infarct volume fraction of < 75% (b)
Table Performance of segmental relative myocardial blood flow (Rel MBF) and perfusable tissue fraction (PTF) by [15O]water in the assessment of myocardial viability.
Copyright American Society of Nuclear Cardiology

## Slide 6
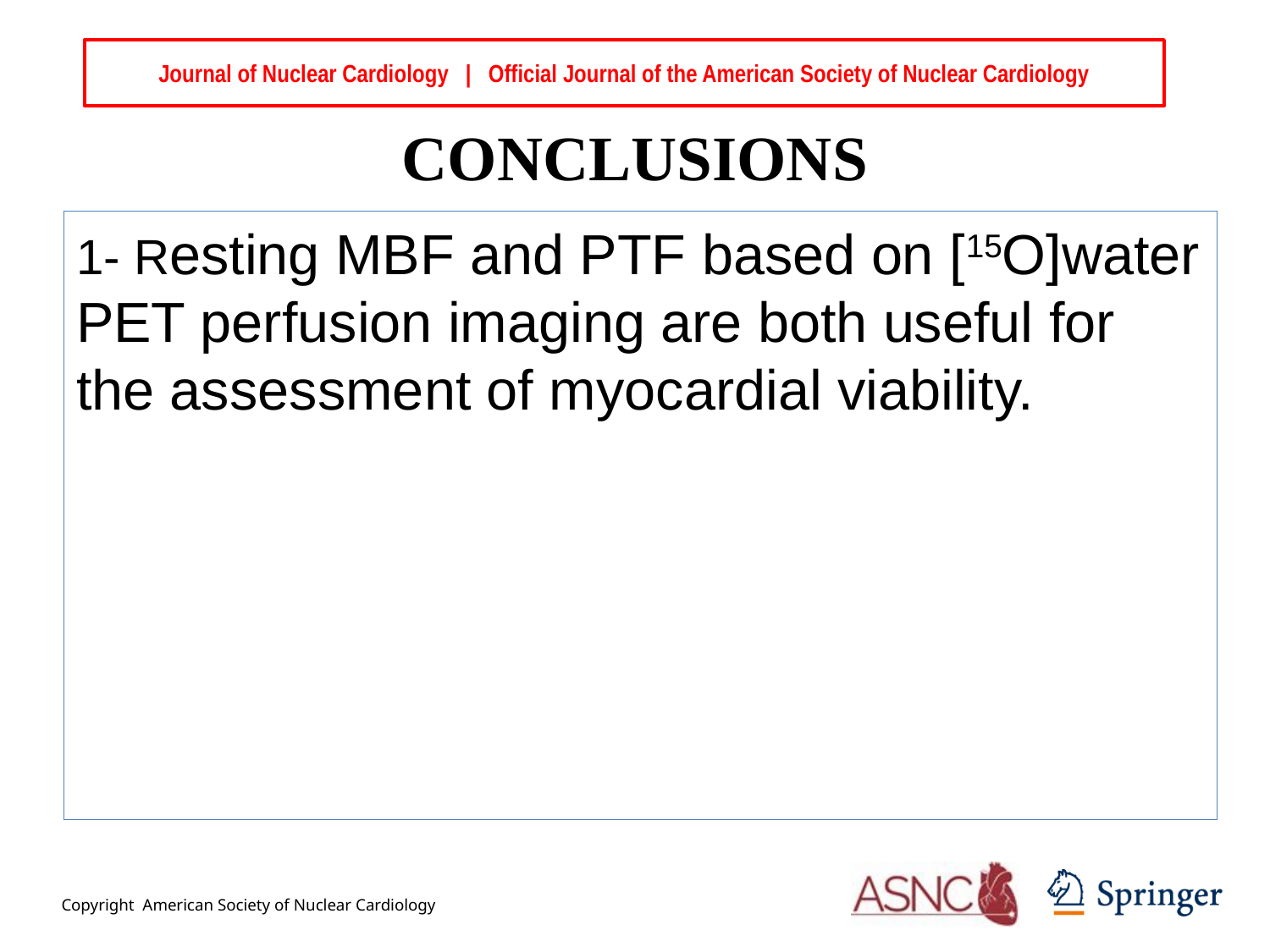

Journal of Nuclear Cardiology | Official Journal of the American Society of Nuclear Cardiology
# CONCLUSIONS
1- Resting MBF and PTF based on [15O]water PET perfusion imaging are both useful for the assessment of myocardial viability.
Copyright American Society of Nuclear Cardiology
