# Supplementary material for: Assessment of myocardial viability with [15O]water PET: A validation study in experimental myocardial infarction
Source: J Nucl Cardiol. 2019 Jul 17;28(4):1271–80. doi: 10.1007/s12350-019-01818-5 (PMC8421281; doi:10.1007/s12350-019-01818-5)
Supplement: Supplementary file 2 — Supplementary material 2 (DOCX 25 kb) [file 12350_2019_1818_MOESM2_ESM.docx]

# Assessment of myocardial viability with [^15^O]water PET: A validation study in experimental myocardial infarction

Maria Grönman, MSc^a^, Miikka Tarkia, PhD^a^, Christoffer Stark, MD, PhD^b^, Tommi Vähäsilta, MD, PhD^b,c^, Tuomas Kiviniemi, MD, PhD^c^, Mark Lubberink, PhD^d,e^, Paavo Halonen, MD^f^, Antti Kuivanen, MD^f^, Virva Saunavaara, PhD^g,h^, Tuula Tolvanen, MSc^g,h^, Jarmo Teuho, PhD^a,g^, Mika Teräs, PhD^g,h^, Timo Savunen, MD, PhD^b^, Mikko Pietilä, MD, PhD^c^, Seppo Ylä-Herttuala, MD, PhD^f,i^, Anne Roivainen, PhD^a,j^, Juhani Knuuti, MD, PhD^a^, Antti Saraste, MD, PhD^a,c,k^

a Turku PET Centre, University of Turku, Turku, Finland

b Research Centre of Applied and Preventive Cardiovascular Medicine, University of Turku, Turku, Finland

c Heart Center, Turku University Hospital and University of Turku, Turku, Finland

d Department of Surgical Sciences, Uppsala University, Uppsala, Sweden

e Department of Medical Physics, Uppsala University Hospital, Uppsala, Sweden

f A.I. Virtanen Institute for Molecular Sciences, University of Eastern Finland, Kuopio, Finland

g Turku PET Centre, Turku University Hospital, Turku, Finland

h Department of Medical Physics, Turku University Hospital, Turku, Finland

i Heart Center, Kuopio University Hospital, Kuopio, Finland

j Turku Center for Disease Modeling, University of Turku, Turku, Finland

k Institute of Clinical Medicine, University of Turku, Turku, Finland

Address for correspondence: Antti Saraste, PET Centre, Turku University Hospital, Kiinamyllynkatu 4-8, FI-20520 Turku, Finland, Email: antti.saraste@utu.fi, Tel: +35823130083

# Supplemental Methods

## Anesthesia and Hemodynamic Monitoring

The animals were anesthetized with midazolam 1 mg/kg (Midazolam Hameln, Hameln Pharmaceuticals GmbH, Hameln, Germany) and xylazine 4 mg/kg (Rompun vet, Bayer Animal Health GmbH, Leverkusen, Germany) intramuscularly (i.m) before the cardiac catheterization, surgical operation and imaging studies. Then, the animals were intubated, connected to a respirator (Dräger Oxylog 3000, Drägerwerk AG, Lübeck, Germany) and ventilated mechanically (tidal volume 8-10 ml/kg, frequency 14-18 breaths per minute). The ear vein was cannulated using a 22G venous catheter and intravenous (i.v.) infusion of propofol 10−50 mg/kg/h (Propofol-Lipuro, B. Braun Melsungen AG, Melsungen, Germany) combined with fentanyl 4−8 µg/kg/h (Fentanyl-Hameln, Hameln Pharmaceuticals GmbH, Hameln, Germany) was used to maintain anesthesia.

The femoral artery was cannulated for monitoring of hemodynamic parameters during imaging studies. Diastolic, systolic and mean arterial pressure and heart rate were recorded using a pressure transducer (TruWave, Edwards Lifesciences, Irvine, CA, USA) that was connected to an anesthesia monitor.

## Stent Implantation

The bottleneck stent was implanted in to the proximal LAD coronary artery of 4 pigs. The method has been described previously [1]. In brief, an introducer sheath (6F, Cordis, Bridgewater, NJ, USA) was placed percutaneously in the femoral artery. GE Innova 3100^IQ^ three-dimensional (3-D) angiography device (GE Healthcare, Waukesha, WI, USA) was used in the catheterization. A sterilized polytetrafluoroethylene tube (diameter 5/64 in.; Fluorplast, Petalax, Finland) with a bottleneck diameter of 0.9 mm was inserted on a Coroflex Blue Ultra (B. Braun Medical; profile 0.8 mm) bare metal stent. The correct positioning and patency of the bottleneck stent in the proximal LAD was confirmed by X-ray fluoroscopy. A Femostop device (St. Jude Medical, St. Paul, MN, USA) was used to secure hemostasis after the removal of the sheath.

Amiodarone 200 mg/day per orally (p.o.) (Cordarone, Sanofi, Paris, France) and bisoprolol 2.5 mg/day p.o. (Bisoproact, Actavis Group, Hafnarfjordur, Island) were administered starting 1 week before the stenting until the end of the study. 750 mg i.v. bolus of Cefuroxime (Zinacef, GlaxoSmithKline, Brentford, UK), 100 mg i.v. bolus of lidocaine (Lidocain, Orion Corporation, Espoo, Finland) and 2.5 ml i.v. bolus of magnesium sulphate (246 mg/ml, Addex-magnesiumsulfaatti, Fresenius Kabi AB, Uppsala, Sweden) were given before catheterization. Acetylsalicylic acid 300 mg p.o. (Primaspan, Orion Corporation, Espoo, Finland) and clopidogrel 300 mg p.o. (Plavix, Sanofi, Paris, France) were given 1 day before stenting. Enoxaparin 30 mg i.v. (Sanofi, Paris, France) was administered after the insertion of an introducer sheath, and another 30 mg was given subcutaneously (s.c.) after removing the sheath and securing hemostasis. Daily doses of acetylsalicylic acid (100 mg/day p.o.), clopidogrel (75 mg/day p.o.), and enoxaparin (30 mg/day s.c.) were continued throughout the study.

## Surgical Operation

Sixteen pigs were operated as previously described [2, 3]. Short left anterior thoracotomy was performed, and distal LAD was completely ligated using a 5-0 monofilament polypropylene suture (Prolene, Ethicon, Norderstedt, Germany). An ameroid constrictor (2.50 or 2.75 mm, model MRI-2.50-TI and MRI-2.75-TI; Research Instruments SW, Escondido, CA, USA) was placed around the proximal LAD approximately 15 minutes after the ligation.

Fentanyl 4-8 µg/kg i.v. was administered intraoperatively and fentanyl 2-4 µg/kg/hour (Matrifen transdermal patch, Takeda Pharma A/S, Roskilde, Denmark) postoperatively for 3-7 days. The thoracotomy wound was anesthetized with locally administered intramuscular injection of bupivacain 25 mg (Bicain, Orion Pharma, Espoo, Finland) at the end of the operation. 30 mg/kg i.v. bolus of cefuroxime (Cefuroxime, Orion Pharma, Espoo, Finland) was administered preoperatively.

Amiodarone (Cordarone, Sanofi-Synthelabo Ltd., Newcastle upon Tyne, UK) was administered 8 mg/kg perorally (p.o.) daily starting 1 week before and lasting for 2 weeks after the surgery. Amiodarone 6 mg/kg i.v., metoprolol 2 mg/kg i.v. (Seloken, Genexi, Fontenay sous Bois, France) and magnesium sulfate (MgSO4) 25 mg/kg i.v. (Addex-magnesiumsulfaatti, Fresenius Kabi AB, Uppsala, Sweden) were administered intraoperatively. Clopidogrel 3 mg/kg p.o. (Plavix, Sanofi Winthrop Industrie S.A., Ambare`s et Lagrave, France) was administered daily starting 1 day before and continuing for 2 weeks after the surgery.

# Supplemental results

**Supplemental Table** Performance of segmental relative myocardial blood flow (Rel MBF), perfusable tissue fraction (PTF) and perfusable tissue index (PTI) by [^15^O]water in the assessment of myocardial infarction and viability.

| Viable tissue (infarct volume fraction < 50%) | | | | | |
| --- | --- | --- | --- | --- | --- |
|  | Sensitivity | Specificity | Positive predictive value | Negative predictive value | Accuracy |
| PTI (cut off ≥ 82%) | 96% | 65% | 90% | 84% | 89% |
| Rel MBF (cut off ≥ 80%) | 78% | 85% | 95% | 53% | 80% |
| PTF (cut off ≥ 66%) | 85% | 75% | 92% | 59% | 82% |
|  |  |  |  |  |  |
| Viable tissue (infarct volume fraction < 75%) | | | | | |
|  | Sensitivity | Specificity | Positive predictive value | Negative predictive value | Accuracy |
| PTI (cut off ≥ 82%) | 95% | 82% | 97% | 74% | 93% |
| Rel MBF (cut off ≥ 67%) | 91% | 86% | 97% | 65% | 90% |
| PTF (cut off ≥ 66%) | 82% | 86% | 97% | 47% | 82% |

# References

1. Rissanen TT, Nurro J, Halonen PJ, Tarkia M, Saraste A, Rannankari M, et al. The bottleneck stent model for chronic myocardial ischemia and heart failure in pigs. Am J Physiol Heart Circ Physiol 2013;305:H1297-308

2. Teramoto N, Koshino K, Yokoyama I, Miyagawa S, Zeniya T, Hirano Y, et al. Experimental pig model of old myocardial infarction with long survival leading to chronic left ventricular dysfunction and remodeling as evaluated by PET. J Nucl Med 201152:761–768.

3. Tarkia M, Stark C, Haavisto M, Kentala R, Vähäsilta T, Savunen T, et al. Cardiac remodeling in a new pig model of chronic heart failure: Assessment of left ventricular functional, metabolic, and structural changes using PET, CT, and echocardiography. J Nucl Cardiol 2015;655–665.
